# Clinical significance of microbiota changes under the influence of psychotropic drugs. An updated narrative review

**DOI:** 10.3389/fmicb.2023.1125022

**Published:** 2023-03-01

**Authors:** Agata Misera, Igor Łoniewski, Joanna Palma, Monika Kulaszyńska, Wiktoria Czarnecka, Mariusz Kaczmarczyk, Paweł Liśkiewicz, Jerzy Samochowiec, Karolina Skonieczna-Żydecka

**Affiliations:** ^1^Department of Psychiatry, Pomeranian Medical University in Szczecin, Szczecin, Poland; ^2^Department of Biochemical Science, Pomeranian Medical University in Szczecin, Szczecin, Poland; ^3^Sanprobi sp. z o.o. sp.k., Szczecin, Poland

**Keywords:** pharmacomicrobiomics, psychotropic drugs, gut, brain, microbiota

## Abstract

Relationship between drugs and microbiota is bilateral. Proper composition thus function of microbiota is a key to some medications used in modern medicine. However, there is also the other side of the coin. Pharmacotherapeutic agents can modify the microbiota significantly, which consequently affects its function. A recently published study showed that nearly 25% of drugs administered to humans have antimicrobial effects. Multiple antidepressants are antimicrobials,. and antibiotics with proven antidepressant effects do exist. On the other hand, antibiotics (e.g., isoniaside, minocycline) confer mental phenotype changes, and adverse effects caused by some antibiotics include neurological and psychological symptoms which further supports the hypothesis that intestinal microbiota may affect the function of the central nervous system. Here we gathered comprehensively data on drugs used in psychiatry regarding their antimicrobial properties. We believe our data has strong implications for the treatment of psychiatric entities. Nevertheless the study of ours highlights the need for more well-designed trials aimed at analysis of gut microbiota function.

## 1. Introduction

The human digestive tract is inhabited by billions of microorganisms forming a specific ecosystem called the intestinal microbiota. It includes bacteria, fungi, archaea and eukaryotes (Gomaa, 2020). Environmental conditions in various sections of the gastrointestinal tract, such as the availability of oxygen and nutrients, the pH value, the speed of passage of chyme, and even the structure and immunological properties of the intestinal epithelium, determine the number and the types of microorganisms that inhabit these parts. Gender and epigenetic factors, such as diet, medications, illnesses, the latitude of residence, age, also affect the formation of the intestinal microbiota, which is individual for each person (Shanahan et al., 2021).

Microorganisms are crucial in digestive processes and affect the immune and metabolic systems. In addition, being one of the elements of the brain-intestinal axis, microbiota affects the functioning of the brain and, thus, emotions and cognitive processes (Cryan et al., 2019; Dalile et al., 2019; Fan and Pedersen, 2021; de Vos et al., 2022).

The results of studies published in 2018 show that nearly 25% of the 1,000 human medicines tested belonging to different therapeutic groups have an antimicrobial effect, which may result in gut microbiota imbalance with all its consequences (Maier et al., 2018). In addition, a new field of knowledge—pharmacobiomics—describes the relationship between the microbiota and drugs (Doestzada et al., 2018). Knowledge in this area reevaluates the way we think about the pharmacokinetics and pharmacodynamics of drugs [8]. Moreover, this interaction should not be regarded only as unfavorable. For instance, it is believed that the microbiota may support the activity of drugs acting on the Central Nervous System (CNS; Cussotto et al., 2021a).

**Figure 1:**
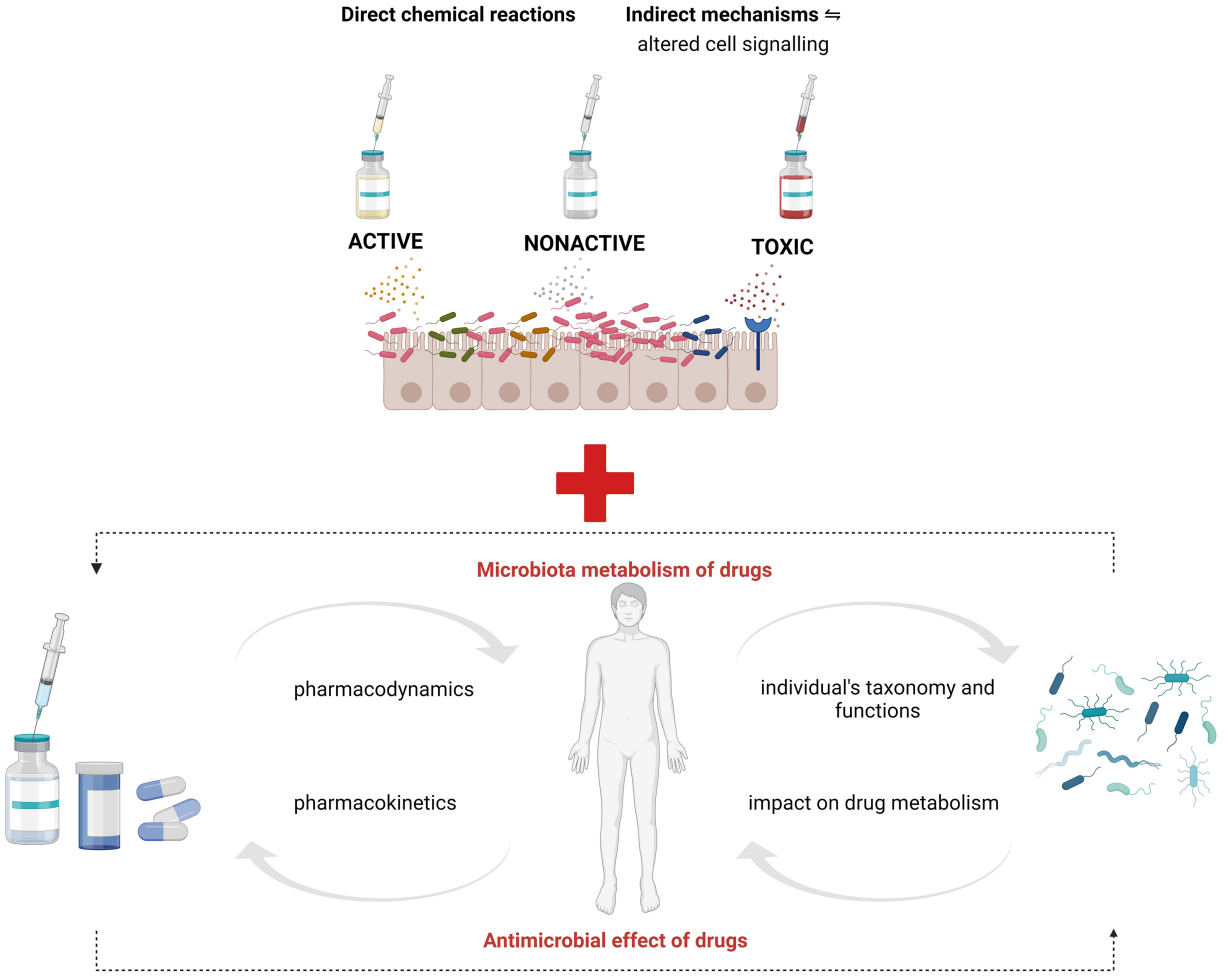
Microbiota interaction with drugs. Based on Tuteja and Ferguson (2019). Metabolic activity of microbiota towards xenobiotics may be direct (e.g., acetylation, denitration, hydrolysis, etc.), which may consistently make a given therapeutic effective or lose its efficacy completely. Indirectly, the metabolites of the intestinal microbiota may compete with drugs for enzymes or receptors, which potentially modulate therapeutic efficacy, also in the mechanisms of altered expression of selected genes involved in intracellular signaling. Parallelly, certain drugs might change the gut environment and promote/inhibit certain bugs bloom which in turn may impact the pharmacokinetics of taken drugs.

The influence of intestinal bacteria on the metabolism of drugs can be directly carried out in bacterial chemical processes (acetylation, deconjugation, dehydration, etc.) or indirectly related to, for example, competition for enzymes or receptors, which results in changes in cell signaling. In addition, after entering the portal circulation to the liver, microbiota metabolites are excreted with bile into the duodenum, which may modulate the effectiveness of therapy or be one of the causes of side effects (Koppel et al., 2017). Finally, the drugs themselves can have antimicrobial effects (Cussotto et al., 2019a; Vich Vila et al., 2020), which in turn may affect their pharmacodynamics and pharmacokinetics (Tsunoda et al., 2021; Figure 1). The best known is the effect of antibiotics on the bacterial composition of the digestive tract. However, many studies also report a significant impact of other commonly used drugs, such as proton pump inhibitors, laxatives, metformin, statins or psychotropic drugs, on the composition and functions of the intestinal bacterial ecosystem (Weersma et al., 2020).

Studies on the impact of psychotropic drugs on the intestinal microbiota are still in the initial phase. They consist, as in the case of other medications, of *in vitro* studies, experimental studies in animals and clinical trials. The data obtained so far are pretty sparse, and their translational significance is minimal (Cussotto et al., 2019a). However, we would like to find an answer to whether these data are currently clinically applicable and can help treat patients with psychiatric diseases, at least to a limited extent. When analyzing the results of previous studies, it should be emphasized that their authors paid great attention to ensure the proper antimicrobial concentrations of drugs tested *in vitro* (Ejim et al., 2011) and that *in vivo* studies were conducted at an excellent methodological level (Cussotto et al., 2021b). Unfortunately, against this background, clinical trials fare worse due to small, often heterogeneous groups of patients and the lack of studies involving repetitive microbiota measurements over a long period of treatment (longitudinal studies). In addition, many studies have focused on describing changes in the composition of the intestinal microbiota under various stimuli, including pharmacological treatment, without attempting to interpret these changes in the clinical context (Cussotto et al., 2021a).

Previous research confirms that the microbiota of people with mental disorders differs from healthy people (Łoniewski et al., 2021). The role of intestinal microbiota in the pathogenesis and clinical course of neurodevelopmental (Garcia-Gutierrez et al., 2020; Cao et al., 2021) and neurodegenerative (Marizzoni et al., 2017; Katz Sand et al., 2019; Aho et al., 2021) disorders is also postulated. Furthermore, it has been proven that endotoxins (lipopolysaccharides) released by pathogenic microorganisms can affect mood and cognitive abilities (Zhao et al., 2019; Evrensel et al., 2020). It is not surprising, therefore, that researchers see the modification of the composition and function of the intestinal microbiota as a new therapeutic possibility (Knudsen et al., 2021; Misera et al., 2021). In addition, the bidirectional interactions of psychopharmaceuticals with the intestinal microbiota are also interesting due to their impact on microecology/macroecology, e.g., by inducing antibiotic resistance (Lu et al., 2022).

This narrative review aims to present the interactions between the drugs most commonly used in patients suffering from psychiatric disorders (except antibiotics, which are described in other publications) and the microbiota. Particular attention will be paid to the clinical significance of these interactions, which will help to understand their impact on the therapeutic process, increase the effectiveness of treatment and help reduce side effects. This study’s five key critical phases were: (1) Identifying the research question: “what is known about the interactions between psychotropic drugs and the gastrointestinal microbiome?.” (2) Identifying relevant studies: PubMed and Embase databases were searched from database inception until 30.09.2022 using the following keywords: microbiota, pharmacology, psychotropic drugs, and pharmacomicrobiomics. We also performed a manual reference search of relevant reviews describing the effects of drugs on the gut microbiome (overall). Finally, we searched for studies in English, with no restrictions regarding publication time. (3) Study selection: we selected studies conducted in both animals and humans, which provided mechanisms on the mode of effects of drugs on the gut microbiome. The first and senior authors performed the study selection. This step was conducted over 2 weeks. (4) Charting the data: we abstracted data on primary outcomes referring to drugs and their effects on the gut microbiota. (5) Collecting, summarizing, and reporting the results: we organized the data thematically according to different types of psychotropic drugs. Finally, we aligned the body of the manuscript with the information on the most recent data on the effects of drugs on the gut microbiome.

## 2. Interaction: stress—microbiota—drugs—mental health

**Figure 2:**
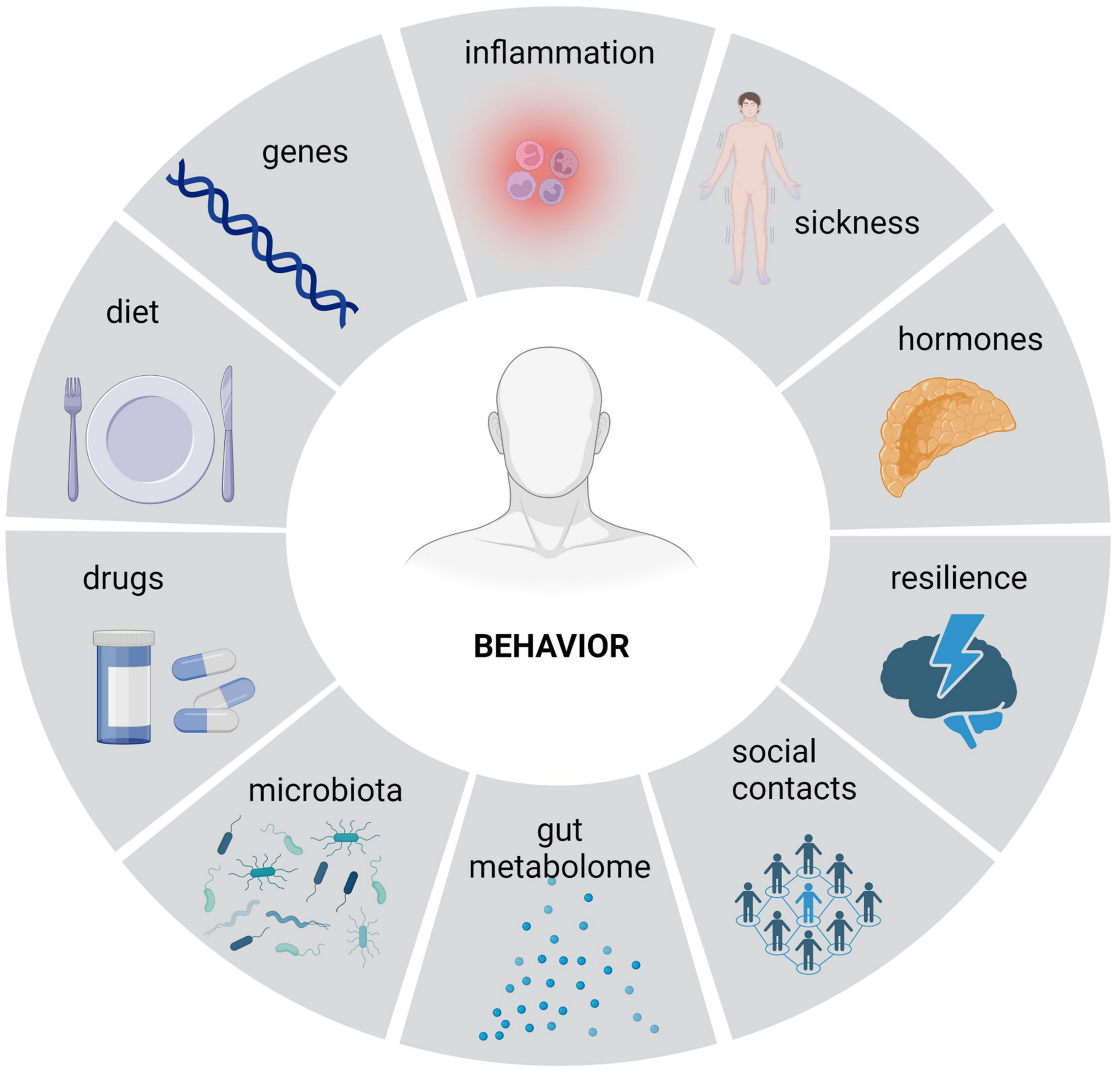
The impact of certain factors on one’s behavior. A variety of factors influence one’s behavior. Apart from a genetic factors, multiple environmental ones, including microbiota along with its metabolites, have the potential to affect behavior.

A patient reporting to a psychiatric office most often seeks help due to behavioral disorders that prevent him from continuing to function at an acceptable level. Behavioral disorders are the primary symptom of mental illnesses. However, their characteristics are determined not only by the specificity of a given disease entity but also by other factors, such as, among others, inflammatory processes, psychosocial factors, the ability to cope with stress, which is often referred to as stress resistance, neuroendocrine factors or the composition of the intestinal microbiota (Sylvia and Demas, 2018; Figure 2).

**Figure 3:**
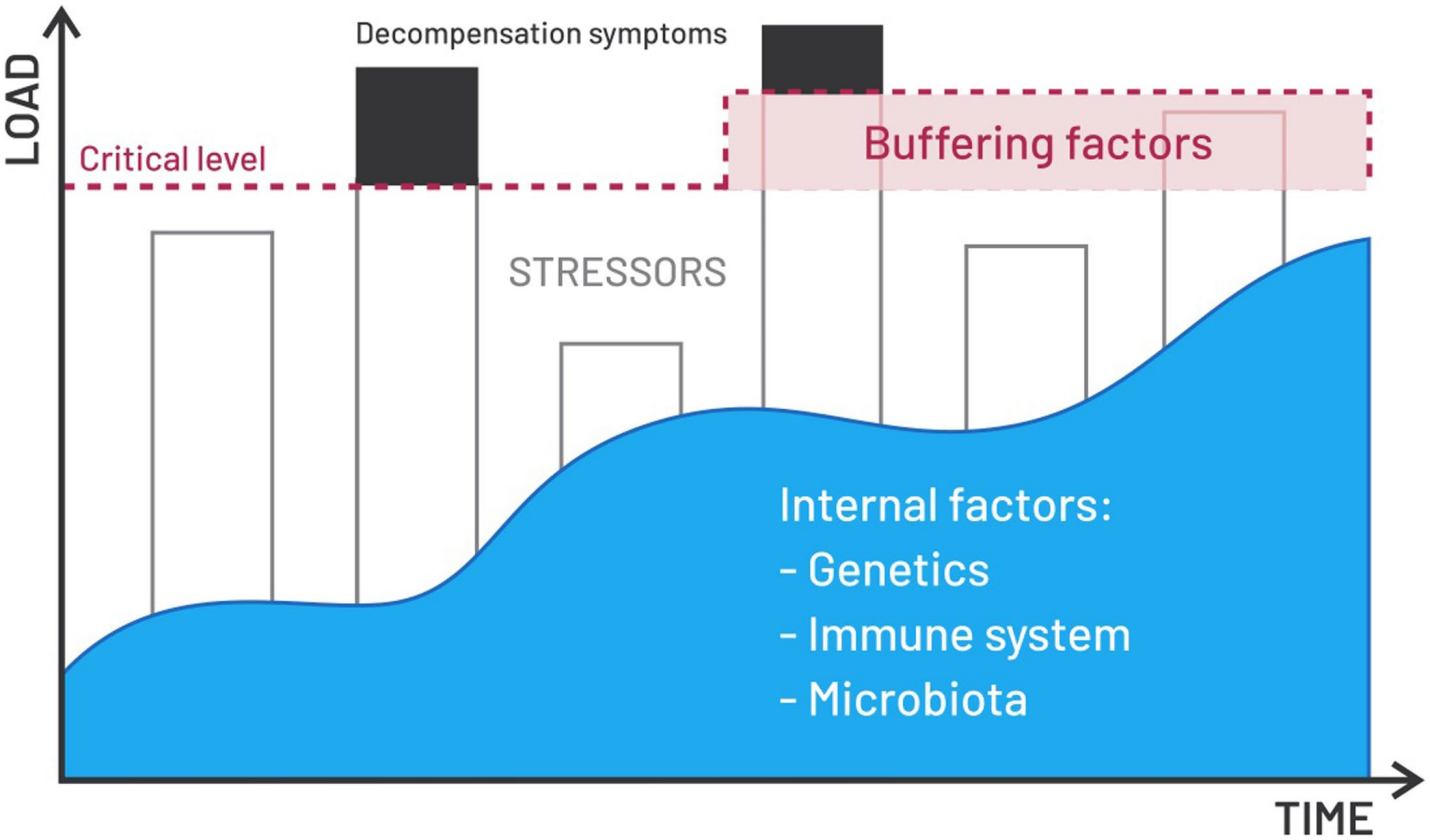
Vulnerability-stress model. This is a model illustrating a psychological theory about the relationship between internal factors, psychosocial factors and vulnerability to stress, which is associated with an increased risk of various diseases, including mental theses. The composition of the gut microbiota appears to be an important internal factor that, through appropriate modulation, can increase individual resistance to stress. Disturbances in the composition of the gut microbiota, are responsible for a number of reactions of the body exposed to stress. Among other things, the gut microbiota influences the concentration of cortisol in the blood. In addition, it participates in the coordination of biochemical reactions in metabolic pathways. These pathways are either stimulated or inhibited, depending on changes in the quantity and quality of the bacterial enterotype. Also, this mechanism at least partially explains the body’s various responses to stress.

Many mental illnesses are manifested by similar behavioral disorders, which is why it is only in a standardized diagnostic process carried out according to specific guidelines that the correct diagnosis, necessary for the implementation of appropriate therapy, can be made. However, it is indisputable that high-stress levels are an inseparable element of any mental illness (Zorn et al., 2017). Not only that but stress is also considered one of the factors leading to the development of mental disorders (Davis et al., 2017). The vulnerability-stress model perfectly illustrates this relationship (Figure 3; Zuckerman, 1999).

This model is one of the leading psychological theories trying to explain the relationship between internal factors (such as genetics, the reactivity of the immune system, and intestinal microbiota), buffering factors, such as good relations with loved ones, a sense of security, a sense of belonging to a group, susceptibility to widely understood stress and the development of mental disorders. Summing up the most important points in developing the vulnerability-stress model, it can be said that the more buffering and stable internal factors a person has, the greater his stress resistance. The vulnerability-stress model perfectly explains why some people are highly resistant to stress despite an objectively measured, significant load, and others experience a nervous breakdown for seemingly trivial reasons. In addition, this model is supported by the theory that people with a genetic load or unfavorable biological conditions and lack buffering factors not only get nervous faster, but their body can and often reacts to stressors affecting them with the development of mental disorders. This concept, therefore, allows for the construction of an individual model of the impact of internal factors, buffering factors and stress intensity on the development and course of mental disorders (Demke, 2022).

The intestinal microbiota is a significant internal factor, which, unlike, for example, genetics, can be effectively modulated in many different ways. Thus, appropriate living conditions, nutrition, physical activity, environment, medications and stimulants affect the microbiota composition (Lynch and Pedersen, 2016). It has been proven that disorders in the composition of the intestinal microbiota are responsible for several reactions of the body exposed to stress (Almand et al., 2022; Cooke et al., 2022). Thus, the intestinal microbiota affects, among others, the concentration of cortisol in the blood (Messaoudi et al., 2011). In addition, it participates in the coordination of biochemical reactions in metabolic pathways (Cryan et al., 2019; Skonieczna-Żydecka et al., 2020a). Studies show that these pathways are stimulated or inhibited depending on changes in the quantity and quality of the bacterial enterotype. This mechanism at least partly explains the body’s diverse responses to stress. E.g. increased appetite vs. lack of appetite, excessive sleepiness vs. insomnia or energy surge vs. exhaustion.

The relationship between stress intensity and changes in the intestinal microbiota composition is an essential part of the brain-gut axis (Molina-Torres et al., 2019). Therefore, since stress plays a vital role in the course of mental illness and, at the same time, has an evident impact on the composition of the intestinal microbiota, it seems important to thoroughly understand the role of the intestinal microbiota in the diagnostic and therapeutic process of mental disorders.

There is a close relationship between the composition and function of the intestinal microbiota, the integrity of the intestinal barrier, well-being, and abdominal discomfort (Skonieczna-Żydecka et al., 2018). Psychiatric patients requiring pharmacological treatment are particularly vulnerable to the latter. A significant problem for patients treated with psychotherapeutics is the side effects of the drugs they take, especially with long-term therapy, the risk of cardiometabolic disorders increases. They are also one of the main reasons for discontinuation of treatment, which is associated with a high risk of recurrence of disease symptoms and, consequently, the disintegration of life (Correll et al., 2018).

## 3. Psychiatric treatment

Despite several data confirming the microbiome’s influence on the bioavailability of drugs, which may have clinical significance (Spanogiannopoulos et al., 2016), there are no such data on psychotropic drugs. Research in this direction is necessary because, in the case of many drugs from this therapeutic group, their effectiveness depends on blood concentration, e.g., “therapeutic window” in the case of lithium, carbamazepine or valproate (Greenberg et al., 2016; Carli et al., 2022). There is no doubt that microbiota should be an element of therapeutic strategies used in personalized medicine (Foster, 2020).

Despite the heterogeneity in psychopharmaceuticals’ chemical composition, most have antibacterial activity (Caldara and Marmiroli, 2021; Table 1) For example, it has been studied that taking antidepressants, especially fluoxetine, amitriptyline, venlafaxine, paroxetine, mirtazapine and trazodone, affecting the composition of the intestinal microbiota (Kalaycı et al., 2014), elevates the risk towards *Clostridioides difficile* infection (Rogers et al., 2013). The relationship between the qualitative and quantitative composition of the intestinal microbiota and the functioning of the brain and CNS is visible on many levels. For example, the antidepressant effect of certain antibiotics from the tetracycline group has been proven (Pae et al., 2008; Schmidtner et al., 2019). In addition, many side effects when taking antibiotics manifest themselves in neurological and mental symptoms [20–22].

**Table 1:** The summary of the impact of psychotropic drugs on microorganisms.

Drug class	Active substance	Decrease	Increase
Antidepressants	Clomipramine	*Leishmania donovani*, *Trypanosoma cruzi* (with benzimidazole), methicillin-resistant *Staphylococcus pseudintermedius*	ND
	Imipramine	*Leishmania donovani*, *E*. *coli*, *Yersinia enterocolitica*, *Giardia lamblia*	ND
	Amitriptyline	*Staphylococcus* spp., *Bacillus* spp., *Vibrio cholera*, *Cryptococcus* spp., *Candida albicans; methicillin-resistant Staphylococcus pseudintermedius*	ND
	Promethazine	*E*. *coli*, *Yersinia enterocolitica*	ND
	Desipramine	*Plasmodium falciparum*, *Lactobacillus casei*, *Akkermansia muciniphila*, *Bacteroides fragilis*	ND
	Sertraline	*E*. *coli*, *S*. *aureus* ATCC 6538, *E*. *coli* ATCC 8739, *P*. *aeruginosa* ATCC 9027, *Aspergillus niger*, *Aspergillus fumigatus*, *Succinivibrio*, *Prevotella*	ND
	Fluoxetine	*L*. *rhamnosus*, *E*. *coli* (including K12 strain), *S*. *aureus*, *P*. *aeruginosa*	ND
	Escitalopram	*E*. *coli*	ND
	Ketamine	*S*. *aureus* (also *MRSA*), *S*. *epidermidis*, *E*. *faecalis*, *S*. *pyogenes*, *P*. *aeruginosa*, *E*. *coli*, *Stachybotrys chartarum*, *Borrelia burgdorferi*, *C*. *albicans* (with antifungal agents)	ND
Antipsychotics	Aripiprazole	ND	*Prevotella*, *Victivallis*, *Desulfovibrionaceae*, *Clostridium*, *Ruminiclostridium*, *Intestinibacter*, *Eubacterium coprostanoligenes*
	Chlorpromazine	*E*. *coli*, *S*. *aureus*, *Proteus mirabilis*, *Klebsiella pneumoniae*	ND
	Fluphenazine	*Salmonella typhimurium*, *Candida glabrata*	ND
	Olanzapine	*E*. *coli NC10*, *Ruminococcus bromii*	ND
	Prochlorperazine	*Bacillus* spp., *Staphylococcus* spp.	ND
	Risperidone	*Alistipes*, *Lactobacillus*, *Akkermansia*	*Bacteroides*, *Allobaculum*, *Turicibacter*, *Aneroplasma*
	Thioridazine	*MRSA*, *Enterococcus* sp., *Mycobacterium tuberculosis*, *Pseudomonas aeruginosa*, *Mycobacterium avium*, *Plasmodium falciparum*, *Trypanosoma spp*., *Salmonella enterica serovar Typhimurium 74*	ND
	Trifluoperazine	*S*. *aureus*, *Shigella spp*., *Vibrio cholerae i V*. *parahaemolyticus*, *Mycobacterium tuberculosis*, *Cryptococcus neoformans*, *E*. *coli*	ND
Analgesics and anticonvulsants	Lamotrigine	*B*. *subtilis*, *S*. *aureus*, *S*. *faecalis*	*Staphylococcus caprae*, *Dorea longicatena*, *E*. *coli*, *Klebsiella aerogenes* (all with carbamazepine)
	Tramadol	*E*. *coli*, *S*. *epidermidis*, *S*. *aureus*, *P*. *aeruginosa*	ND
	Methadone	*S*. *aureus*, *P*. *aeruginosa and S*. *marcescens*, *A*. *muciniphila*	ND

ND - no data.

### 3.1. Antidepressants

Antidepressants are used not only in the treatment of depression but also play an essential role in treating anxiety and obsessive–compulsive disorders. In addition, an exciting discovery is a fact that antidepressants also have antibacterial properties (Kalaycı et al., 2014). Consistently, therefore, recently published work shows that increased use of duloxetine, sertraline, fluoxetine and bupropion at appropriate doses - in the transformation mechanism- promotes the increased prevalence of antibiotic resistance genes (Lu et al., 2022).

*Tricyclic* antidepressants (TCA) have so far been tested in animal models. Clomipramine and imipramine are cytotoxic to human parasitic protozoa, i.e., *Leishmania donovani* (Mukherjee et al., 2020) and *Leishmania major* (Zilberstein and Dwyer, 1984). In combination with benznidazole, clomipramine is active against *Trypanosoma cruzi* (García et al., 2016). A solution of amitriptyline hydrochloride with bactericidal activity against 253 bacterial strains (72 gram-positive and 181 gram-negative) and five fungal strains was also developed. Moreover, when used in rodents infected with the pathogenic strain *Salmonella typhimurium* NCTC 74, the drug showed antimicrobial activity against the pathogens *Staphylococcus* spp., *Bacillus spp*., *Vibrio cholera*, *Cryptococcus spp*. and *Candida albicans* (Mandal et al., 2010). It has also been demonstrated that amitriptyline and clomipramine are active against methicillin-resistant *Staphylococcus pseudintermedius* (Brochmann et al., 2016). Promethazine and imipramine, on the other hand, showed an inhibitory effect against *E*. *coli* and *Yersinia enterocolitica* by interfering with plasmid replication (Csiszar and Molnar, 1992), and imipramine itself was active against *Giardia lamblia* (Weinbach et al., 1992). On the other hand, desipramine is effective against *Plasmodium falciparum* (Basco and le Bras, 1990) but also *Lactobacillus casei*, *Akkermansia muciniphila* and *Bacteroides fragilis* (Ait Chait et al., 2020).

Selective serotonin reuptake inhibitors (SSRIs) work against *Brucellae* and are synergistic in combination with antibiotics against *Corynebacterium urealyticum* (Muñoz-Bellido et al., 1996). SSRIs also inhibit mucus production in coagulase-negative staphylococci (Cussotto et al., 2019a). After analyzing the antimicrobial activity of the four SSRIs against *E*. *coli*, sertraline was the most potent antibacterial agent, but this was not clinically significant against Gram-negative bacteria (Bohnert et al., 2011). It was additionally confirmed that the growth of *S*. *aureus* ATCC 6538, *E*. *coli* ATCC 8739 and *P*. *aeruginosa* ATCC 9027 after plating with sertraline, especially in combination with antibiotics, is inhibited. In fungi, growth inhibition affected *Aspergillus niger* and *A*. *fumigatus* (Ayaz et al., 2015) Fluoxetine has a strong dose-dependent antimicrobial effect against *L*. *rhamnosus* and *E*. *coli*, including K12 strain (Jin et al., 2018) while escitalopram has only a minor antimicrobial effect on *E*. *coli*. In an *in vivo* experiment, it was also confirmed that fluoxetine and escitalopram significantly increase intestinal permeability in the ileum (Cussotto et al., 2019b). Fluoxetine is active against *S*. *aureus*, standard and resistant strains of *P*. *aeruginosa* and *E*. *coli*, especially in combination with gentamicin and erythromycin (Karine de Sousa et al., 2018; Batista de Andrade Neto et al., 2019; Dos Santos et al., 2020). Recent studies indicate that administration of fluoxetine in an *in vitro* model of sepsis inhibits the synthesis of interleukin 1 beta (IL-1β), interleukin 6 (IL-6), tumor necrosis factor alpha (TNF-α), myeloperoxidase (MPO) activity, monocyte chemoattractant protein – 1 (MCP-1), high sensitivity C-reactive prhs-CRP, procalcitonin (PCT), lactate, and the oxidative stress index (OSI), and di-sulfide levels (Cakir et al., 2022). In a 2019 study, it was confirmed that escitalopram oxalate inhibits the growth of *E*. *coli* at a concentration of 45, 15, and 5 mM, while *Bacillus subtilis* is not inhibited (Valipour et al., 2019). Escitalopram oxalate and clonazepam inhibited multidrug-resistant clinical isolates and standard bacterial strains from the American Type Culture Collection in an *in vitro* study, especially in combination with ciprofloxacin and sulfamethoxazole-trimethoprim. The mechanism of bacterial inhibition by clonazepam is the digestion of plasmid DNA (da Rosa et al., 2021). Exposure of bacteria to fluoxetine and sertraline solutions for 30 days has also been shown to promote the development of antibiotic resistance (Gurpinar et al., 2022). This observation is supported by other studies showing that fluoxetine and amitriptyline may increase the prevalence of aminoglycoside (*aph3iii*A), multidrug (*mdt*K, *mdt*P, *mdt*H, *mdt*G, *acr*A), and tetracycline (*tet*M) resistance genes in the rats model (da Rosa et al., 2021).

Ketamine, whose mechanism of antidepressant action is not fully understood, also exhibits *in vitro* antibacterial activity against six different bacterial strains: *S*. *aureus*, *S*. *epidermidis*, *E*. *faecalis*, *S*. *pyogenes*, *P*. *aeruginosa* and *E*. *coli*, with *S*. *aureus* and *S*. *pyogenes* being the most sensitive. It is worth emphasizing that the culture of these organisms in this study was carried out using propofol - a compound that stimulates the growth of bacteria (Begec et al., 2013). Ketamine at a concentration of 2.49–3.73 mM inhibits the growth of methicillin-resistant *S*. *aureus* (MRSA; Coutinho et al., 2021), and *in vitro* model, it has activity against *Stachybotrys chartarum*, *Staphylococcus epidermidis* and *Borrelia burgdorferi* (Torres et al., 2018). In combination with fluconazole and itraconazole, it is active against *C*. *albicans* resistant to class azoles (de Andrade Neto et al., 2020).

*In vivo* studies using the supply of antidepressants are not numerous. In a recent study, Lukić et al. (2019) administered fluoxetine, escitalopram, venlafaxine, duloxetine or desipramine to lab animals and analyzed the drug-induced changes at the microbiota level using next-generation sequencing (NGS). After analyzing the results, it was found that alpha diversity and richness decreased in the test animals, and at the species level, the number of *Ruminococcus*, *Adlercreutzia*, and an unclassified *Alphaproteobacteria* decreased. When *Ruminococcus flavefaciens* or *Adlercreutzia equolifaciens* were transplanted into animals treated with fluoxetine, it was noticed that the first bug abolished the antidepressant effect of the drug. RNA analysis showed that *R*. *flavefaciens* stimulated changes in gene expression in the cerebral cortex, including downregulation of neuroplasticity genes and upregulation of oxidative phosphorylation genes in mitochondria. In turn, in mice exposed to mild stress (Zhang et al., 2021), administration of fluoxetine and amitriptyline resulted in a decrease in the Firmicutes/Bacteroidetes ratio, with fluoxetine having a tremendous potential to cause these changes. In turn, the increase in the *Bacteroidaceae* was associated primarily with amitriptyline. Both drugs significantly affected the counts of *Parabacteroides*, *Butyricimonas*, and *Alistipes*. At the metagenomic level, these drugs have been shown to alter the metabolic pathways involved in carbohydrate catabolism, membrane transport and signal transduction. This new direction of research gives hope for more effective treatment of psychiatric patients, but also for helping those patients who until now belonged to the group of people resistant to psychopharmacological treatment. Thanks to the better effectiveness of the administered drug, it is possible to lower its dose, reducing the risk of side effects.

Human studies are scarce. Liśkiewicz et al. (2019) observed that a six-week inpatient treatment with escitalopram in an acute depressive episode resulted in an increase in alpha diversity in the fecal microbiota, but a causal relationship with patients’ mental health was not proven. A reanalysis of these results using other bioinformatics and statistical methods did not confirm these observations (Liśkiewicz et al., 2021). Similarly, in the study by Lin et al., no effect of escitalopram (29 days, 10 mg/d) on the composition of the microbiota in patients with major depressive disorder (MDD) was found (Lin et al., 2017). These observations indicate the need to conduct further research in this area and, above all, standardize the microbiome analysis methodology and the statistical methods used to evaluate the results obtained. Bharwani et al. (2020), in a study conducted in a small group (*n* = 15) of patients with MDD, observed that although the administration of citalopram or escitalopram did not affect the composition of the microbiota during the 6-month treatment, but the microbiota differed between those who responded to the therapy and those who did not improve clinically. This may suggest a link between the microbiota and treatment response.

Microbiota modification with probiotics is a very promising method of supporting antidepressant treatment. Nikolova et al. (2021), in their updated review and meta-analysis of 404 depressed patients, found that psychobiotics were effective in reducing depressive symptoms when administered as an adjunct to antidepressant therapy (SMD = 0.83, 95%CI 0.49–1.17) but did not show benefit in monotherapy (SMD = 0.02, 95%CI 0.34–0.30). Potential mechanisms may be associated with an increase in the concentration of brain-derived neurotrophic factor (BDNF) and a decrease in the concentration of C-reactive protein (CRP) under their usage. Results of another meta-analysis, including 603 patients (Misera et al., 2021), showed the influence of psychobiotics on psychometric tests in patients with depression. However, the effect of psychobiotics was not of great clinical importance and was effective in combination with other antidepressants, which was confirmed in the meta-analysis of Nikolova et al. (2021). It should be emphasized that taking probiotics is well tolerated, safe and does not pose a risk in patients with MDD.

Additionally, this intervention is not associated with significant side effects (Misera et al., 2021). In mechanistic studies, Rudzki et al. (2019) and Kazemi et al. (2019a,b) observed an improvement in tryptophan metabolism due to probiotic therapy. Akkasheh et al. (2016) and Reiter et al. (2020) observed the anti-inflammatory effects of probiotics, which, however, have not been confirmed in other studies (Romijn et al., 2017; Rudzki et al., 2019). Heidarzadeh-Rad et al. (2020) reported an increase in the level of BDNF, which correlated with the response to antidepressant treatment. However, the meta-analysis did not confirm the effectiveness of the use of probiotics in the regulation of parameters related to the HPA axis (cortisol level), inflammation (interleukins, tumor necrosis factor—TNF) and the tryptophan degradation pathway (kynurenine). In another meta-analysis, Amirani et al. (2020) reported that the intake of probiotics by patients with various mental disorders (not only MDD) had a beneficial effect on CRP, IL-10 and malondialdehyde (MDA) levels but did not affect other markers of inflammation (TNF-alpha, IL-1B) and oxidative stress. However, due to the ambiguous results of the studies and the high heterogeneity of the studied populations, it can be said that the clinical mechanism of action of probiotics in improving the symptoms of MDD remains the subject of speculation.

It is known that the effect of probiotics/psychobiotics is strain-dependent. However, the meta-analyses conducted so far have not confirmed the effectiveness of a particular strain in patients with MDD. Interestingly, the administration of probiotics is associated with a better clinical response (Misera et al., 2021) and improves patients’ cognition. However, it has not been shown that the effect of probiotics is always associated with the impact on the intestinal microbiota composition, although it may determine its functions (Liśkiewicz et al., 2019). Similar observations apply to metabolic diseases, in which the microbiota does not seem to change its composition but the functions of the microbiome (Kaczmarczyk et al., 2022). Finally, it is worth noting that one study observed that gut barrier integrity and markers of inflammation (gut microbiota can affect both of these parameters) might be related to the response to treatment in MDD patients and the severity of symptoms (da Rosa et al., 2021). The observed changes, however, were of a correlation rather than a cause-and-effect relationship (Łoniewski et al., 2021).

### 3.2. Antipsychotics

**Figure 4:**
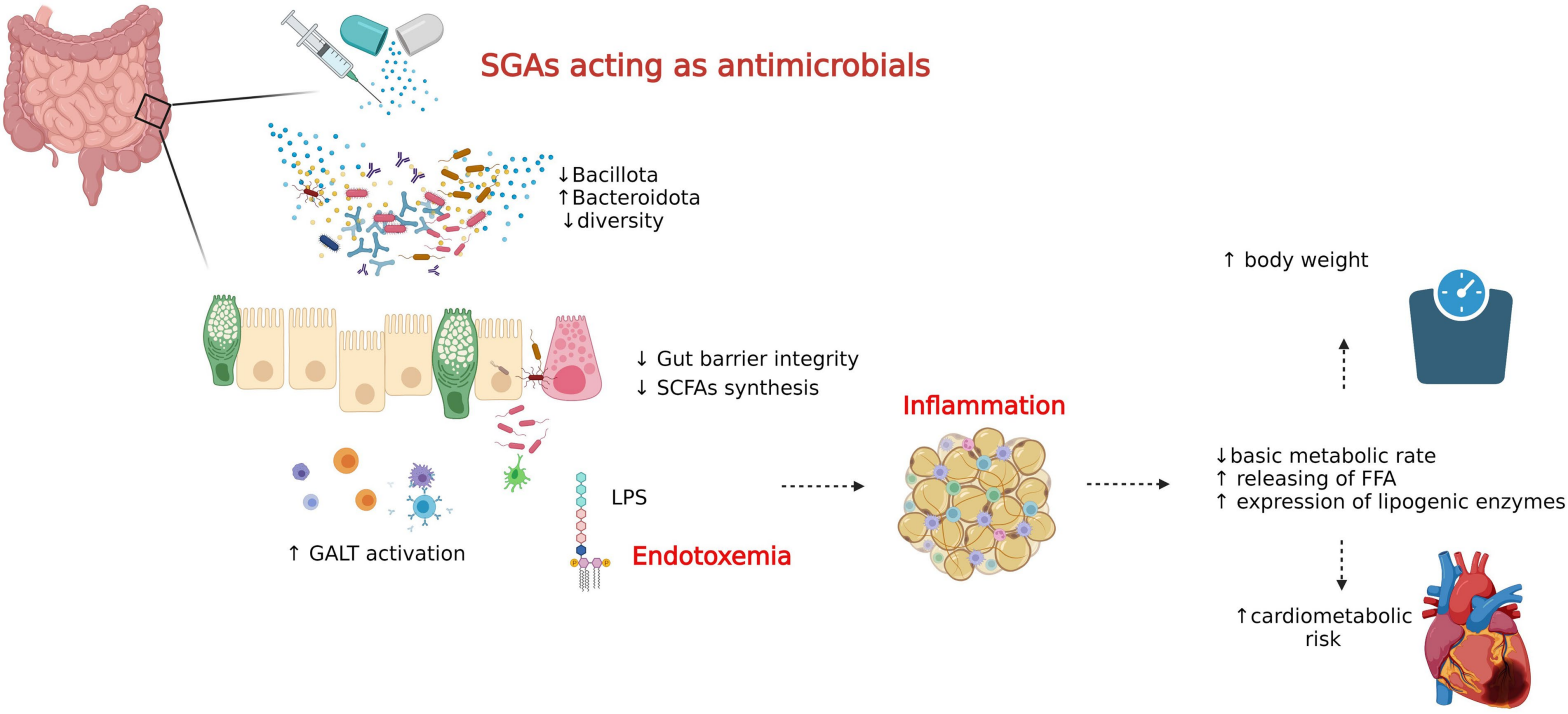
Microbiotic and cardiometabolic effects of second-generation antipsychotics. Schematic presentation of possible mechanisms responsible for metabolic disorders secondary to SGA treatment. Based on Skonieczna-Żydecka et al. (2019) and Singh et al. (2022). Metabolic disturbances observed in the course of antipsychotics administration may be caused by adverse changes in the composition of gut microbiome - changes in the abundance of major types of gut microbes which consequently disturb gut barrier integrity and SCFAs pool. This might phenotypically manifests as weight gain, hypertriglyceridemia, hypercholesterolemia, hypertension and impaired glucose metabolism and the mechanisms involved include lowering basal metabolic rate, enhancement of lipogenic enzymes expression and releasing of free fatty acids.

Antipsychotics are, next to antidepressants and sedatives, the most commonly used drugs in psychiatry. Taking into account the antimicrobial properties of antipsychotic drugs, it seems that the metabolic disorders observed in the course of therapy with these drugs may be caused by unfavorable changes in the composition of microorganisms responsible for metabolic activity (Skonieczna-Żydecka et al., 2019). Indeed, clinical studies and animal studies have shown that treatment with Second Generation Antipsychotics (SGAs) induces significant changes in the abundance of the main types of intestinal microorganisms, resulting in weight gain, hypertriglyceridemia, hypercholesterolemia, hypertension and glucose metabolism, which significantly increases the risk of developing metabolic syndrome and cardiovascular events (Davey et al., 2012, 2013; Bahr et al., 2015a,b; Flowers et al., 2017; Riedl et al., 2017; Kao et al., 2018). Furthermore, the results of the latest scoping review, in which 46 studies were analyzed, showed that antipsychotic drugs were causing disturbances within the intestinal barrier, including intensification of gut-associated lymphoid tissue (GALT) activity and reduction of short-chain fatty acids (SCFA) synthesis, are associated with the induction of metabolic changes observed with such therapy (Singh et al., 2022; Figure 4).

Microbiota analysis of 76 hospitalized elderly patients by Ticinesi et al. (2017) showed that the use of antipsychotic drugs was strongly associated with the composition of the gut microbiome. An increased abundances of *Prevotella*, *Victivallis* and *Desulfovibrionaceae* species were observed during treatment. In an *in vitro* study by Cussotto et al. (2019b) it was found that administration of aripiprazole (ARI) significantly increased the richness and diversity of microbial species. At the genus level, several species belonging to *Clostridium*, *Peptoclostridium*, *Intestinibacter* and *Christensenellaceae* were increased after ARI treatment. Moreover, increased permeability in the ileum was recorded. Ma et al. (2020) observed the difference in the composition of the intestinal microbiota between a group of patients with schizophrenia who did not take medication and those treated with antipsychotics. Yuan et al. (2021) observed an increase in α-diversity and decreased *Lachnoclostridium*, and increased *Romboutsia* abundance in schizophrenia (SCH) patients after 24 weeks of risperidone (RIS) treatment. In addition, treatment response was significantly associated with basal levels of these bacteria.

The influence of the type of antipsychotic drugs on the microbiota has not been confirmed by Nguyen et al. (2021) in a well-designed and analyzed study that took into account the compositional nature of the microbiota and used machine learning. However, the results were affected by the data’s multidimensional nature and the study’s cross-sectional nature.

Long-term use of antipsychotics is associated with side effects, including significant weight gain, particularly with olanzapine (OLZ) and clozapine, moderate with quetiapine and risperidone (RIS), and lowest with ARI (Dayabandara et al., 2017). Such adverse metabolic effects also apply to children and adolescents in whom antipsychotics are used (Bretler et al., 2019).

Morgan et al. (2014), Davey et al. (2012, 2013), and Bahr et al. (2015a,b), in their experimental work, observed that weight gain during treatment with OLZ and RIS is closely related to the composition of the intestinal microbiota. Davey et al. reported that the co-administration of olanzapine and an antibiotic in rats attenuates the metabolic effects associated with olanzapine use (Davey et al., 2013). Kao et al. (2018) proved that the prebiotic galacto-oligosaccharides (B-GOS) significantly reduced body weight gain in rats treated with OLZ, while not affecting the drug’s effect. A recent study by Luo et al. (2021) showed that olanzapine given to rats for 35 days increased the Firmicutes:Bacteroidetes ratio. At the species level, OLZ treatment reduced the relative abundance of *Ruminococcus bromii* compared to controls. Phenotypically, the drug’s effect resulted in an increase in body weight, glucose concentration and unfavorable changes in lipid metabolism, as well as an increase in adipocyte volume and fatty liver. These symptoms subsided after the introduction of metformin - a drug inhibiting weight gain (Hu et al., 2014), which probably also causes microbiota changes (Silamiķele et al., 2021). Consequently, metformin restored microbiota homeostasis, although at the level of the intestinal ecosystem, there was a further reduction in the number *Lactobacillus reuteri* and *Bacteroides pectinophilus*, and also the growth of the abundance of *Bacteroides uniformis*, *Bacteroides acidifaciens*, *Ruminococcus bromii*, *Desulfovibrio simplex*, and *Arcobacter butzleri* (Luo et al., 2021).

Other side effects include adverse effects on glucose tolerance, increased cholesterol and triglyceride levels, and hypertension. These symptoms very often lead to the development of metabolic syndrome in patients (De Hert et al., 2011), and its prevalence is close to 30% (Sanchez-Martinez et al., 2018). Flowers et al. (2017) investigated the interactions between treatment and the gut microbiota in a cross-sectional study of 117 patients with bipolar disorder. Forty-nine people were subjected to SGA therapy, while 68 did not take any medications, constituting the control group. It has been noted that taking medications is associated with changes in the composition of the intestinal microbiota. The number of *Lachnospiraceae*, *Akkermansia and Sutterella* genera were changed. The number of *Lachnospiraceae* bacteria increased in the SGA group, while the number of *Akkermansia* decreased. Such a pattern was also noted in obese patients who were not using antipsychotics.

A cross-sectional study of 18 boys aged 8–15 years taking RIS was conducted by Bahr et al. (2015a). The control group included 10 patients aged 10–14 with psychiatric disorders who were not taking SGA. Chronic use of RIS led to an increase in the subjects’ body mass index (BMI) and a significantly lower ratio of Bacteroidetes to Firmicutes compared to the control group. Li et al. (2021) observed that after 24 weeks of RIS treatment, BMI, glucose, Homeostatic Model Assessment (HOMA-IR), Total-cholesterol, low-density lipoprotein cholesterol (LDL-C), high-density lipoprotein cholesterol (HDL-C) and triglyceride levels were significantly changed compared to pre-treatment values. It was also found that the baseline abundance of *Christensenellaceae* and *Enterobacteriaceae* was significantly associated with changes in triglycerides, BMI and HOMA-IR after 24 weeks of RIS treatment. Similarly, Yuan et al. (2018) found that 24-week administration of RIS causes significant metabolic disorders and changes in the composition of the microbiota, among which changes in the abundance of *Bifidobacterium* spp. were correlated with changes in body weight.

Significant changes in the composition of the intestinal microbiome during 6 weeks of treatment with OLZ in patients with acute schizophrenia were not observed by Pełka-Wysiecka et al. (2019). The study involved 20 hospitalized patients taking daily doses of 5 to 20 mg of OLZ. Analysis of the intestinal microbiota of patients with SCH has shown that it is highly individual in taxonomy and functionality. In addition, it does not change during the 6-week therapy. Two types of enterotypes of the intestinal microbiome in patients with SCH were distinguished: type 1 was characterized by a predominance of *Prevotella* bacteria, while type 2 was characterized by a high amount of *Bacteroides*, *Blautia* and *Clostridium*. Pharmacotherapy was associated with clinical improvement in all patients and significant weight gain in women. The severity of symptoms at the beginning of therapy varied depending on the expected metabolic activity of the microbiota (Pełka-Wysiecka et al., 2019).

First-generation antipsychotics (FGA) also have antimicrobial properties. Thioridazine, a phenothiazine drug, has *in vitro* antimicrobial activity against methicillin-sensitive strains *of S*. *aureus* (Ordway et al., 2002; Hahn and Sohnle, 2014; Tozar et al., 2020), resistant to vancomycin: *Enterococcus* (Wainwright et al., 1999), *Mycobacterium tuberculosis* (Ordway et al., 2003; Amaral and Viveiros, 2017), *Pseudomonas aeruginosa* and *Mycobacterium avium* (Viveiros et al., 2005; Ruth et al., 2020). In addition, it has been shown to inhibit the growth of *Plasmodium falciparum* and *Trypanosoma* spp. (Hrynchuk and Vrynchanu, 2019), and also *Salmonella enterica serovar Typhimurium* 74 (Dasgupta et al., 2010). Fluphenazine has a pronounced activity against Gram-positive and Gram-negative bacteria, including *Salmonella typhimurium* (Dastidar et al., 1995). Recent results indicate that this drug protects against *Candida glabrata infection* due to inhibiting calmodulin [128]. Trifluoperazine strongly inhibited the growth of *Shigella* spp., *Vibrio cholerae* and *Vibrio parahaemolyticus* at concentrations of 10–100 μg/ml (Mazumder et al., 2001) but has also been shown to be active against replicating, non-replicating and refractory *Mycobacterium tuberculosis* (REDDY et al., 1996; Advani et al., 2012), and also *Cryptococcus neoformans* (Wang and Casadevall, 1996). Chlorpromazine, in turn, has anti-mycobacterial properties *in vitro* (Crowle et al., 1992) and restricts the growth of *S*. *aureus* and *E*. *coli*.(Coutinho et al., 2008). A recent study gave the possibility to use chlorpromazine-enriched catheters to prevent biofilm formation by *Escherichia coli*, *Proteus mirabilis* and *Klebsiella pneumoniae* in urinary tract infections (Sidrim et al., 2019). In one of the few and at the same time the most recent studies, Manchia et al. (2021) assessed the effect of both SGA and FGA on the microbiome of patients with SCH. At the type level, Bacteroidetes and Fusobacteria increased in FGA users, and at the family level, *Fusobacteriaceae*, *Streptomycetaceae*, *Helicobacteraceae*, and *Bacillaceae*. *Clostridiales* Family XII *Incertae Sedis* were not observed in this group of patients in contrast to the group treated with SGA. In the group taking typical antipsychotics, bacteria *Fusobacterium*, *Butyricimonas*, *Blautia*, *Paraprevotella*, *Klebsiella* were more numerous. At the species level, this group has increased counts of *Parabacteroides merdae*, *Ruminococcus* sp. AT10, *Clostridium piliforme*, *Enorma massiliensis*, *Megamonas rupellensis*, *Bacteroides ovatus*, *Butyricimonas* sp. GD2 and *Ruminococcus* sp. DJF VR70k1. At the same time, they were generally absent in patients taking SGA.

### 3.3. Analgesics and anticonvulsants

For other drugs used in psychiatry, such as anticonvulsants or opioid analgesics, the literature is scarce. Lithium and valproate do not inhibit *E*. *coli* and *L*. *rhamnosus in vitro*. However, in rodents, they can significantly change the microbiota inhabiting the caecum, causing changes in the number of *Ruminococcaceae* and *Bacteroides* (Cussotto et al., 2019b). Valproate used in patients with epilepsy for 3 months did not cause statistically significant changes in the microbiota, including diversity indices, but at the end of the observation, an increase in the Firmicutes/Bacteroidetes ratio was observed (Gong et al., 2022). One recent study also showed that valproate stimulates the production of trans-9-elaidic acid in *S*. *ludwigii* TBRC2149, a biomolecule previously found to inhibit beta-oxidation in macrophages (Poolchanuan et al., 2020). In turn, the latest research by Huang S, et al. (2022) showed that lithium carbonate used in a model of intestinal inflammation induced beneficial changes in the microbiota by normalizing diversity, an increase in the abundance of *A*. *muciniphila* and an increase in the synthesis of SCFA. In addition, in lamina propria, it stimulated the activation of anti-flooded regulatory T cells.

TREG cells (Huang S, et al., 2022). Lamotrigine has antibacterial activity against Gram-positive bacteria: *B*. *subtilis*, *S*. *aureus* and *S*. *faecalis* (Journal of Chemical Sciences | Indian Academy of Sciences, n.d.). Together with carbamazepine, it caused toxic effects in intestinal epithelial cells *in vitro* and inhibited the growth of several species of bacteria representing a healthy digestive system in children up to 9 years of age (such as *Ruminococcus gnavus*, *Clostridium ramosum*, and *Roseburia intestinalis*) and patients with drug-resistant epilepsy (including “anti-epileptic” *Parabacteroides species*), while the growth of *Staphylococcus caprae*, *Dorea longicatena*, *E*. *coli*, and *Klebsiella aerogenes* growth was enhanced (Ilhan et al., 2022). Tramadol has strong *in vitro* bactericidal activity against *E*. *coli* and *S*. *epidermidis* and weak antimicrobial activity against *S*. *aureus* and *P*. *aeruginosa* (Tamanai-Shacoori et al., 2007), and methadone against S. *aureus*, *P*. *aeruginosa* and *S*. *marcescens* (Sheagren et al., 1977). Tramadol administered at concentrations of 12.5 and 25 mg/ml to BALB mice infected with *Staphylococcus aureus* and *Pseudomonas aeruginosa* induced a reduction in wound diameter (by inflammation induction and phagocytic activity) caused by *S*. *aureus* (Farzam et al., 2018). After the administration of morphine, changes in the composition of the gut microbes in rodents were associated with a significant increase in the number of pathogenic bacteria and a decrease in stress tolerance communities (Wang et al., 2018). In a cohort of patients with cirrhosis, chronic opioid use was associated with a reduction in the relative abundance *of Bacteroidaceae and Ruminococcaceae* (Acharya et al., 2017). Cruz-Lebron et al. examined the microbiota in an opioid-dependent mouse model (Cruz-Lebrón et al., 2021). Repeated intraperitoneal treatment with methadone resulted in a decrease in *A*. *muciniphila* numbers and, consequently, in the synthesis of SCFA. In another study patients received opioid agonists (heroin, prescription opioids), antagonists (naltrexone), a combination agonist–antagonist (buprenorphine-naloxone), or no medication. Those using opioid agonists (no antagonists) had lower microflora diversity, *Bacteroides* enterotype, and lower relative abundance of *Roseburia*, a butyrate-producing genus, and *Bilophila*, a bile acid metabolizing genus (Gicquelais et al., 2020).

## 4. Discussion

So far, much evidence has been collected that the composition of the intestinal microbiota is specific in mentally ill people compared to persons with no such diseases (Chen et al., 2021). These observations lead to the hypothesis that the composition of the microbiota correlates with the mental state of the subjects. Cussotto et al. (2021a) hypothesized that the modulation of the composition of the intestinal microbiota by psychiatric drugs should not be treated only as an adverse effect but as an integral part of their activities aimed at the symptoms of mental diseases. In their research, they noticed that drugs with different chemical structures (such as lithium, valproate or aripiprazole) modified the composition of the intestinal microbiota in a very similar way. The growth of a similar niche of intestinal bacteria was observed during therapy (Cussotto et al., 2019b). Interestingly, Maier et al. (2018) made similar observations when studying a group of antipsychotics. These results also suggest that the antimicrobial effect of psychiatric drugs should be considered not as a side effect of the drugs but rather as one of the mechanisms of action. This would mean that psychopharmaceuticals, on the one hand, leading to changes in the composition of the intestinal microbiota, may induce cardiometabolic disorders, but on the other hand, these changes contribute to the reduction of clinical symptoms. Therefore, it seems that the intestinal microbiota not only influences a person’s resistance to stress and the risk of developing mental disorders but also plays an important role in the therapeutic process, directly and indirectly influencing the metabolism of drugs, and probably modulating the intensity of mental symptoms. Intestinal microbiota may therefore turn out to be the key to personalized psychiatric treatment of patients, perfectly fitting into the ideas of holistic patient care. Especially in the case of mental illness, which is affected by so many biological and psychosocial factors, an individual therapeutic approach is extremely important.

**Figure 5:**
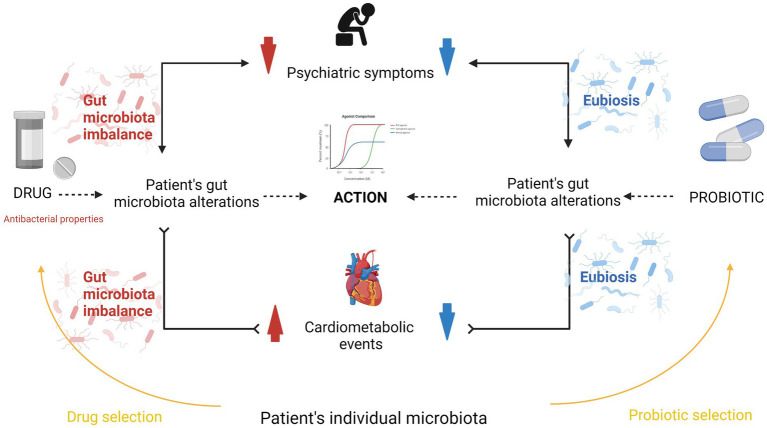
The influence of microbiota on the effectiveness of treatment in psychiatry. A mentally ill patient has a specific composition of intestinal microbiota. This composition differs from that of the intestinal microbiota of the healthy population. Psychopharmacotherapy, for example, with antipsychotic drugs, which, in addition to the known mechanism of action, that is, among other things, blockade of dopamine D2 receptors, also has a bactericidal effect by changing the composition of the intestinal microbiota of the patient. The alleviation of clinical symptoms of mental illness may depend not only on the known pharmacological effects of psychotropic drugs, but also on little-known interactions of these drugs with the intestinal microbiota. These interactions may also have another face. With long-term treatment, metabolic disorders resulting from the administration of antipsychotic drugs are observed, which may be related to microbiota disorders. Severe metabolic disorders are one of the most common reasons for discontinuing psychopharmacological treatment or forcing doctors to change the drug, despite its good effect on the symptoms of the psychiatric disorder. Supplementation with appropriate probiotic strains, especially those that affect the brain-gut axis, can significantly alleviate the discomfort associated with metabolic disorders and at the same time support the positive effects of the drug on the patient.

Bearing in mind the above-mentioned circumstances, it is necessary to consider the practical significance of interactions between the intestinal microbiota and drugs used in psychiatry. The following areas come to the fore (Figure 5):

1. Influence of the composition/function of the microbiota before treatment or its changes during treatment on 1a/ efficacy and 1b/ side effects of psychotropic drugs,
2. Impact of microbiota modification on the effectiveness of drugs used in psychiatric treatment.

So far, the predictive value of microbiota analysis in the selection or effectiveness of antipsychotic treatment seems to be very distant. Research in this direction requires standardization and validation. The same applies to the role of microbiota as a marker of the effectiveness and safety of antipsychotic treatment.

Definitely, more hope should be associated with the use of probiotics, which in this case should be defined as psychobiotics. Psychobiotics are live bacteria that, when administered in adequate amounts, benefit the mental health of the host (Dinan et al., 2013). In order to demonstrate such an effect, it is necessary to demonstrate their effectiveness in a randomized clinical trial (RCT) and to ensure certain quality requirements (the right amount of CFU/AFU bacteria throughout the product’s shelf life; Binda et al., 2020). Jamilian and Ghaderi (2021) in a RCT, double-blind, placebo-controlled study lasting 12 weeks, found that a probiotic (LactoCare^®^, Zisttakhmir Company, Tehran, Iran) containing *Lactobacillus acidophilus*, *Bifidobacterium* (*lactis*, *bifidum*, and *longum*) at a dose of 8 × 10^9^ CFU/day +200 mcg/day selenium significantly improved overall PANSS scores compared to placebo, had antioxidant and anti-inflammatory effects, and reduced insulin resistance. The authors did not analyze the composition of the microbiota. Kang et al. (2021) conducted an RCT involving patients with the first episode of psychosis who had not used AP for at least 3 months prior to study entry. Patients were randomized to treatment with OLZ (15–20 mg/day) or OLZ plus a probiotic preparation (Bifico, triple live bacteria oral capsule) consisting of *Bifidobacterium*, *Lactobacillus* and *Enterococcus* at 5.0 × 10^7^ CFU/g for 12 weeks. Taking the probiotic reduced fasting insulin and HOMA-IR compared to the group treated with OLZ alone. Statistically insignificant decreases in BMI were also observed. The composition of the microbiota was also not analyzed. Severance et al. (2017) observed a reduction in *Candida albicans* IgG antibody titers (only males) after 14 weeks of administration of *L*. *rhamnosus* GG (10^9^ CFU) and *Bifidobacterium Animals subsp*. *lactis* Bb12 (10^9^ CFU). No effect of the probiotic on the PANSS score was observed, and sero-negativeness for *C*. *albicans* was correlated with improvement in positive symptoms and bowel motility. Yang et al. (2021), in a 12-week study involving patients with schizophrenia or schizoaffective disorder treated with olanzapine, observed the effect of *Bifidobacterium*, *Lactobacillus*, and *Enterococcus* bacteria containing 1 × 10^7^ CFU of each strain in a capsule, on parameters such as body weight, BMI, and appetite latencies. After 4 weeks of treatment, the group taking the probiotic together with OLZ had lower weight gain and BMI. At 8 and 12 weeks, no difference was recorded between patients in both groups. Taking the probiotic also had no effect on appetite latencies. Tomasik et al. (2015), in a randomized, double-blind, placebo-controlled study, found that a probiotic containing *Lactobacillus rhamnosus* GG (10^9^ CFU) and *Bifidobacterium animalis* subsp. *lactis* Bb12 (10^9^ CFUs) administered to patients with schizophrenia, or schizoaffective disorders did not contribute to a decrease in the intensity of schizophrenia symptoms as measured by the Positive and Negative Syndrome Scale (PANSS) score, but significantly decreased acute-phase reactant von Willebrand factor and increased the levels of MCP-1, BDNF, T-cell specific protein Regulated upon Activation, Normal T cell Expressed and presumably Secreted; RANTES, Macrophage inflammatory protein 1 beta. In addition, *in silico* analysis suggested that probiotics regulated the immune functions *via* IL-17 pathway. Dickerson et al. (2014) conducted an RCT involving patients with moderately severe psychotic symptoms according to the PANSS scale. Although the supply of the probiotic *Lactobacillus rhamnosus GG* (10^9^ CFU) and *Bifidobacterium animalis subsp*. *lactis* Bb12 (10^9^ CFUs) for 14 weeks did not contribute to the improvement of psychotic symptoms, but patients supplemented with probiotic complained about significantly fewer gastrointestinal complaints. Huang J, et al. (2022) in RCTs evaluated the effect of probiotic (1,680 g/d: *Lactobacillus*—3.8 × 10^8^ CFU/g, *Bifidobacterium*—1.7 × 10^9^ CFU/g, and *Enterococcus*—7.8 × 10^8^ CFU/g), dietary fiber (60 g/d) and their combination on antipsychotic-induced weight gain in 136 patients with severe psychiatric disorders, of whom 118 completed the study. It was observed that the combination of probiotics and dietary fiber resulted in significant weight loss. Probiotics or dietary fiber alone could prevent weight gain, while significant weight gain was observed in the placebo group. In addition, probiotics and/or dietary fiber had a beneficial effect on insulin sensitivity, which was not observed in the placebo group. In addition, it has been shown that taking a probiotic along with fiber has the most significant impact on the diversity and composition of intestinal bacteria. Logistic regression analysis showed that greater microbiota richness was positively correlated with weight loss.

Microbiota changes may also be important when antipsychotics are combined with metformin (Correll et al., 2020; Wang et al., 2021). Although no studies have been conducted on this subject, it is worth remembering that the use of metformin may also have a positive effect on the growth of metabolically beneficial bacteria, such as *A*. *muciniphila*, *Escherichia* spp. or *Lactobacillus* spp., while reducing the number of other, potentially unfavorable, e.g., *Intestinibacter* (MetaHIT consortium et al., 2015; Rodriguez et al., 2018). Importantly, *A*. *muciniphila* has a positive effect on metabolism in many ways (Plovier et al., 2017). And one of its proteins - Amuc1100 - regulates glucose metabolism and is used in the treatment of obese and diabetic patients, improving clinical parameters such as insulin sensitivity, body weight, the content of fat mass and non-functional enzymes of plasma of hepatic origin (Depommier et al., 2019). In addition, the number of *A*. *muciniphila* increases in direct proportion to the number of goblet cells. There are also studies showing that the use of this substance has a positive effect on the pool of produced metabolites, including SCFAs or insulinotropic peptides (Rodriguez et al., 2018). Additionally, *A*. *muciniphila* acts in CNS (Cheng et al., 2021; Higarza et al., 2021; Sun et al., 2022). It is therefore postulated that the reconstruction of the microbiota altered by a metabolic disease under the influence of metformin may be functionally beneficial, but in some patients - especially during the period of increasing the dosage - it may cause gastrointestinal disorders (Nabrdalik et al., 2022). The observed side effects may be related to the effect of the drug on the microbiota (MetaHIT consortium et al., 2015; Elbere et al., 2018, 2).

The cited data show that the interaction of antipsychotic drugs—microbiota—metformin can be very complex, and only well-designed clinical trials will allow to optimize and individualize such therapy. Metformin administration is of interest not only for the treatment of metabolic disorders caused by SGA treatment (Skonieczna-Żydecka et al., 2020b), in which dysbiosis plays a significant role (Skonieczna-Żydecka et al., 2019) but above all, the possibility of a beneficial effect of this drug for mental disorders (Dodd et al., 2022).

Interactions between drugs used in patients with mental disorders and the microbiota, as well as methods of its modulation, are confirmed in many experimental and clinical studies. They may be associated with side effects, but they also give hope for increasing the effectiveness of psychiatric treatment and reducing its complications. Therefore, it is necessary to include the study of these interactions in long-term clinical trials and to conduct mechanistic and translational analyses.

## Author contributions

KS-Ż, AM, and IŁ gave conception to the study. AM, IŁ, MoK, PL, WC, MaK, and JP conducted the research. AM, IŁ, PL, KS-Ż, and JS contributed to analysis and interpretation of data. KS-Ż, AM, and IŁ drafted the paper and substantively revised it. JS and KS-Ż supervised the work. All authors contributed to the article and approved the submitted version.

## Conflict of interest

KS-Ż and MaK receive remuneration from Sanprobi, Szczecin, Poland—manufacturer of probiotics, and IŁ is a shareholder of this company. However, the content of this study was not constrained by this fact.

The remaining authors declare that the research was conducted in the absence of any commercial or financial relationships that could be construed as a potential conflict of interest.

## Publisher’s note

All claims expressed in this article are solely those of the authors and do not necessarily represent those of their affiliated organizations, or those of the publisher, the editors and the reviewers. Any product that may be evaluated in this article, or claim that may be made by its manufacturer, is not guaranteed or endorsed by the publisher.
